# Editorial: Morphogens in the Wiring of the Nervous System

**DOI:** 10.3389/fncel.2015.00502

**Published:** 2016-01-08

**Authors:** Juan P. Henríquez, Nelson Osses

**Affiliations:** ^1^Laboratory of Developmental Neurobiology, Department of Cell Biology, Faculty of Biological Sciences, Millennium Nucleus of Regenerative Biology, Universidad de ConcepcionConcepción, Chile; ^2^BMP Research Group, Faculty of Sciences, Institute of Chemistry, Pontificia Universidad Católica de ValparaísoValparaíso, Chile

**Keywords:** morphogen, Wnt proteins, BMP, Shh, nervous system

Morphogens are secreted signaling molecules that play instructive roles to regulate tissue patterning and cell identity in a concentration-dependent fashion during early embryonic development. Four principal mammalian morphogens include retinoic acid, as well as members of the Wingless-int (Wnt) and transforming growth factor beta (TGF-β)/bone morphogenic protein (BMP) and sonic hedgehog (Shh) families. Morphogen gradients control a plethora of developmental events related to specification and differentiation, cell and tissue polarity, growth control and regeneration.

In the nervous system, neuronal differentiation, the polarized outgrowth of neuronal projections, the pathfinding of axons toward their target cells, and the recognition of pre- and post-synaptic partner cells are fundamental requisites to allow the assembly of functional synapses. During the last decades, a growing body of evidence gathered from invertebrate and vertebrate model organisms has shown that the same morphogens classically known to orchestrate early embryonic development are also involved in the precise wiring of the nervous system.

The aim of this research topic is to highlight the fundamental roles that morphogens play during the establishment of synaptic connectivity. Hence, we have brought together 12 original research articles and eight reviews. They are mainly focused on Wnt (Aviles et al.; Bernis et al.; Berwick and Harvey; Dickins and Salinas; Pinto et al.; Rosso and Inestrosa; Silva-Alvarez et al.; Varela-Nallar and Inestrosa; Aviles et al.; Varela-Nallar et al.), BMP (Gamez et al.; Pinto et al.; Osses and Henriquez), and Shh (Aviles et al.; Reinchisi et al.) signaling pathways. Research also covers the function of signaling cascades activated by other types of morphogens, including the fibroblast growth factors (FGF; Lee and Umemori; Paradiso et al.), the hepatocyte growth factor (HGF) (Bhardwaj et al.), and netrin (Yamagishi et al.). In addition, researchers have contributed with the emerging roles of new molecules, such as the thyroid hormone (Dezonne et al.), SCO-spondin (Vera et al.), and vitamin C (Pastor et al.). Articles are focused on a wide variety of cellular processes involved in the establishment of neuronal connectivity, such as neurogenesis, neuronal specification, and maturation (Berwick and Harvey; Dezonne et al.; Gamez et al.; Pastor et al.; Reinchisi et al.; Rosso and Inestrosa; Varela-Nallar and Inestrosa; Vera et al.; Varela-Nallar et al.; Yamagishi et al.), axonal outgrowth, polarization, and guidance (Aviles et al.; Bernis et al.; Bhardwaj et al.; Pinto et al.; Aviles et al.; Osses and Henriquez), and synapse formation (Dickins and Salinas; Rosso and Inestrosa; Aviles et al.; Osses and Henriquez), either in physiological contexts or in models of diseases affecting the normal function of the nervous system, including epilepsy (Lee and Umemori; Paradiso et al.), Alzheimer's disease (Silva-Alvarez et al.; Varela-Nallar et al.), and amyotrophic lateral sclerosis (Pinto et al.).

We are confident that this integrative research topic emphasizes the central and pleiotropic roles played by morphogens during neural development. We therefore hope that the original articles and reviews presented here will inspire future directions of research focusing on the diversity of cell signaling mechanisms controlling the assembly, maintenance and regeneration of the nervous system.

## Author contributions

JH and NO wrote, edited and revised the manuscript.

### Conflict of interest statement

The authors declare that the research was conducted in the absence of any commercial or financial relationships that could be construed as a potential conflict of interest.

